# Unusual spread of cervical adenocarcinoma to the endometrium and left fallopian tube: a case report and literature review

**DOI:** 10.1097/MS9.0000000000002153

**Published:** 2024-05-15

**Authors:** Zahraa M. M. Zeer, Duha Jawaada, Sami Bannoura, Saadah Jaber

**Affiliations:** a Faculty of Medicine, Al-Quds University; b Department of Obstetrics and Gynecology; c Department of Pathology, Al-Makassed Hospital, Jerusalem, Palestine

**Keywords:** case report, cervical adenocarcinoma, endometrium adenocarcinoma, immunohistochemistry, misdiagnosis

## Abstract

**Introduction:**

The incidence of cervical adenocarcinoma and adenocarcinoma in situ are gradually increasing especially in younger women. However, unusual spread of cervical adenocarcinoma has rarely been reported.

**Case presentation:**

The authors report a case of a 60-year-old woman who presented with postmenopausal bleeding. She was misdiagnosed to have endometrial adenocarcinoma on the lower uterine segment depending on curettage specimen. After hysterectomy, it was revealed depending on morphological features in histology accompanied with immunohistochemistry that the patient had cervical adenocarcinoma with endometrial and left fallopian tube extension.

**Discussion:**

Distinguishing endocervical adenocarcinoma from endometroid adenocarcinoma poses many challenges especially when evaluating endometrial curettage specimens. Histological diagnosis based on morphological features combined with a panel of immunohistochemistry stains is crucial for accurate diagnosis and identifying the primary origin of the tumor.

**Conclusion:**

Accurate distinction between cervical adenocarcinoma versus endometrial adenocarcinoma is important because of its significant effects on choosing the appropriate treatment option.

## Introduction

Highlights

- To avoid misdiagnosis of cervical adenocarcinoma as endometrial adenocarcinoma, combining the histological features with the immunohistochemistry is required.
- It is possible to misdiagnose cervical adenocarcinoma as endometrial adenocarcinoma while analyzing curettage tissues.
- Unusual spread of cervical adenocarcinoma has been rarely reported compared to cervical squamous cell carcinoma.

Cervical carcinoma is the fourth most common gynecological malignancies^1^. It is divided into squamous cell carcinoma (SCC) and adenocarcinoma. Adenocarcinoma is classified according to its association with human papillomavirus (HPV) infection to endocervical adenocarcinoma HPV-associated and endocervical adenocarcinoma HPV-independent. The former represents the vast majority and its most common type is called the usual type and the most common type of the later one is gastric-type mucinous adenocarcinoma^2^. These two types pose multiple challenges for distinguishing them from other variants. Although, there are many morphological features to aid in the diagnosis, identifying the origin of the primary tumor is impossible without the use of immunohistochemistry panel especially in evaluating endometrial curettage specimens. In this case, we will emphasize the significance for accurate distinction between cervical adenocarcinoma and endometrial endometroid adenocarcinoma. In addition, we will highlight the possibility of misdiagnosis of such cases based on endometrial curettage specimens^3^.

## Case presentation

A 60-year-old woman presented with postmenopausal vaginal bleeding for 1 year. She experienced menopause at age 52 and she has no family history of gynecological malignancies. She is G8P8, all of her children were delivered by uncomplicated normal vaginal delivery and her last delivery was 23 years ago. Her previous gynecological history was unremarkable. When she first sought medical advice, she was diagnosed by her gynecologist to have a cervical polyp 2 cm size. She was advised to do dilation and curettage to investigate the cause of her postmenopausal bleeding. Histologically, she was diagnosed to have moderately differentiated endometrial adenocarcinoma (stage 1A) most likely arising from the polyp with unremarkable cervical biopsy. A whole-body CT scan was recommended, her chest and abdomen CT were unremarkable and her pelvic CT showed distended uterine cavity, filled with fluid with fine endometrial enhancement. Otherwise, her pelvic CT was unremarkable. The patient then was referred to a tertiary care hospital where she underwent total abdominal hysterectomy with bilateral salpingo-oophorectomy.

**Figure 1:**
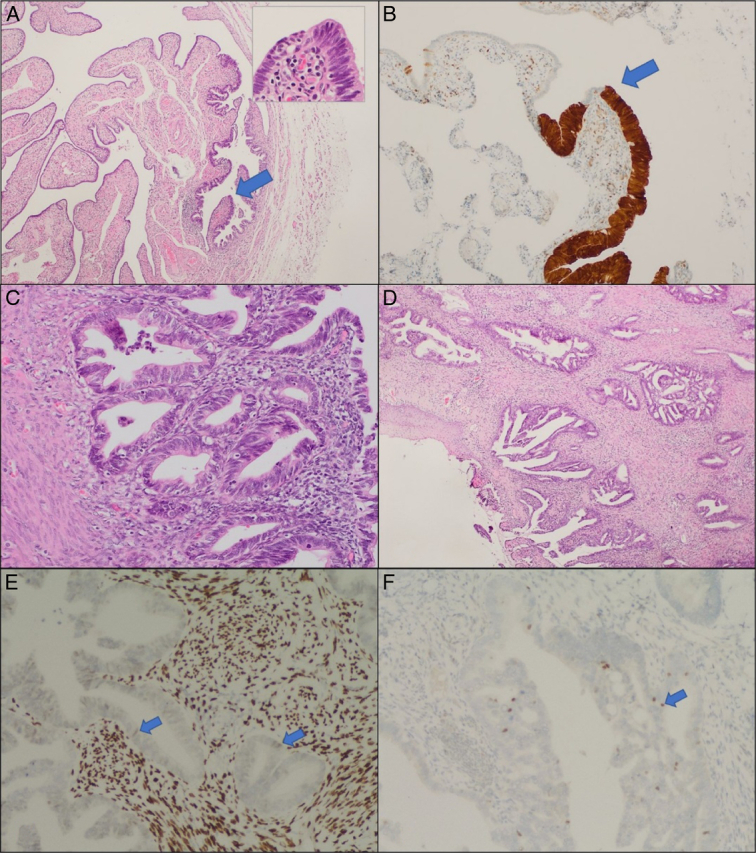
Human papillomavirus (HPV) related endocervical adenocarcinoma with pagetoid spread into endometrium and fallopian tube; A. Section shows cross-section of fallopian tube with area of increased epithelial thickness/crowding (arrow, right third) (H&E, 4X); The insert shows abrupt transition between normal fallopian tube lining (left half) and the malignant endocervical columnar glands (right half); B. The tumor cells in the fallopian tube are diffusely positive for P16 immunostain a surrogate marker for HPV (10X); C. The endometrium is also replaced by the same malignant endocervical glands (H&E, 20X); D. Invasive endocervical adenocarcinoma, HPV related (H&E, 4X). P16 immunostain (not shown) is diffusely positive in the endocervical adenocarcinoma, while estrogen receptor (ER) is focally positive (E) and P53 is wild-type (F).

No lesions were identified grossly and no lymph nodes were found during lymph nodes exploration. Histologically, the specimen was received in formalin. It consists of hysterectomy specimen composed of 8×4×2.5 cm uterus with attached bilateral adnexa consisting of 2.5×2×1 cm for the right ovary, 1.7×1.2×0.9 cm for the left ovary, 5×0.4 cm for the right tube, and 4.5×0.4 cm for the left tube. The endometrium is 0.1 cm thick and the myometrium is 1 cm thick. Under microscope, cross-section of fallopian tube shows area of increased epithelial thickness/crowding with abrupt transition between normal fallopian tube lining and the malignant endocervical columnar glands. The tumor cells in the fallopian tube are diffusely positive for P16 immunostain a surrogate marker for HPV immunostaining, focally weak positive for estrogen receptor, negative for vimentin and shows P 53 as wild type. The endometrium is also replaced by the same malignant endocervical glands (Fig.1). Accordingly, the patient was diagnosed with endocervical adenocarcinoma with endometrial and left fallopian tube extension. This case was written according to the SCARE (Surgical CAse REport) criteria 2023^4^.

## Discussion

Cervical carcinoma affects 500 000 women worldwide^5^ and it is considered the fourth most common gynecological malignancies^1^. SCC is the most common histological subtype and constitutes about 70–80% of all cervical cancer cases^6^ while endocervical adenocarcinoma constitutes about 10–25% from all cases of cervical cancer^1^. Even with the most recent improvement in the Pap smear screening program that lower the incidence of SCC, the incidence of cervical adenocarcinoma and adenocarcinoma in situ are steadily rising in comparison to SCC and as absolute numbers, particularly in younger women^7^. This could be attributed to many factors like difficulty in diagnosing glandular lesions via Pap smear, obesity, nulliparity, and changes in oral contraceptives^8^.

Depending on the HPV association, there are two main types of cervical adenocarcinoma. The most prevalent form of HPV-associated type is thought to be endocervical adenocarcinoma of the usual type, whereas gastric-type is the most common form of HPV-independent cervical adenocarcinoma^2^.

Usual type endocervical adenocarcinoma patients account for 75% of all the cases of adenocarcinoma of the cervix. The average range of patient is between 22 and 80 years and^7^ an average age at presentation is around 50 years^9^. HPV especially 16 and 18 are identified in 80–100% of cases of usual type^7^.

The most common sequence of cervical cancer progression is locoregional stromal invasion with destructive and infiltrative spread. It begins within the cervix, invades downward to the vagina, invades to the parametria, and then invades anteriorly to the bladder or posteriorly to the rectum. In some rare cases, tumor growth can be intraepithelial without stromal invasion and spreads proximally to the endometrium and more rarely to the adnexa^10^. This unusual pattern of growth of cervical cancer makes it challenging to distinguish it from endometrial endometroid adenocarcinoma. Accurate differentiation is not only for academic reasons, it is crucial for correct treatment decisions including type of hysterectomy, lymph node sampling or dissection extent and adjuvant chemotherapy and radiotherapy^11^.

Both the endometrial cancer and cervical cancer can spread through the internal uterine orifice. While endometrium cancer may spread to the cervical canal and cervical stroma, cervical cancer can spread to the endometrial cavity and myometrium. As a result, clinical examination alone is unreliable due to its limited diagnostic accuracy in determining the origin of the primary tumor^12^.

Histological difficulties in diagnosis could be attributed to many causes which include: first, both types of tumors shared the same architecture and growth patterns which could be as glandular or villoglandular. Second, they both could have the same cellular differentiation with endometroid-like and mucinous features^2^. Third, it is not necessarily to consider the dominant tumor as the primary site as some endocervical adenocarcinoma could have dominant endometrial involvement mimicking a primary endometrial adenocarcinoma with extension to the endocervix. Fourth, it is difficult to interpret the type of tumor when it involves the endocervix and the endometrium especially in the lower uterine segment based on a small tissue sample by biopsy and curettage^13^. In this situation, precursor lesions could be overgrown by one cancer or simulate one another (e.g. complex atypical hyperplasia could be stimulated by endocervical adenocarcinoma extension to the endometrium). In these case, misclassification of endometrial adenocarcinoma with cervical extension are reported^14^.

Nevertheless, there are multiple histological features that can help distinguish endocervical adenocarcinoma from endometroid endometrial adenocarcinoma. A higher degree of nuclear atypia, which includes a moderate to severe pleomorphism, hyperchromatic nuclei with sharp contours and coarse chromatin, apically situated mitotic figures and basically situated apoptotic bodies are more likely to be seen in endocervical adenocarcinoma^13^. Other histological features that support the diagnosis of endocervical adenocarcinoma are the presence of coexistent cervical intraepithelial neoplasia (CIN) or the presence of adjacent cervical glandular intraepithelial lesions^15^. On the other hand, squamous morules which are the foci of squamous differentiation are more frequently encountered in endometroid endometrial adenocarcinoma and they are absent in endocervical adenocarcinoma^3^. In addition, mild cellular atypia, round to oval nuclei with pale vesicular chromatin and nucleoli, the presence of stromal foam, coexisting atypical endometrial hyperplasia, less frequent mitotic activity and apoptosis all correlate with the diagnosis of endometroid endometrial adenocarcinoma^15^. In equivocal cases, when both the corpus and the cervix are involved identifying the primary site of tumor by evaluating curettage or hysterectomy specimens may be impossible without the use of a panel of immunostaining which includes P16, estrogen receptor, progesterone receptor, vimentin, and carcinoembryonic antigen^14^.

P16 is a cyclin dependent kinase (CDK) 4 inhibitor that binds to cyclin D-CDK 4/6 complexes which control the G1-S interphase at the cell cycle by inactivating cyclin dependent kinase that phosphorylate retinoblastoma protein (Rb). In preneoplastic and neoplastic lesions that are associated with HPV, E7 protein will inactivate Rb. In this situation, p16 will accumulate as Rb normally inhibits the transcription of p16 protein. As a result, diffuse p16 positivity in most cases is associated with high-risk HPV infection. As molecular technique facilities needed to identify HPV are not available in most laboratories, p16 immunohistochemistry is of value as it is easy to perform^16^. To give additional discriminatory power in some cases, we use methods to detect high-risk HPV in the tumor genome by polymerase chain reaction or in situ hybridization^17^. P16 positivity in a focal or diffuse pattern in cervix or other tissues are reported in non-HPV mechanism that is why we should use p16 as a part of panel that includes vimentin, estrogen receptor, progesterone receptor, and carcinoembryonic antigen^18^.

Vimentin is an intermediate filament protein that shows expression in normal and neoplastic endometrial epithelial cells in immunohistochemistry studies. In the endocervical glandular epithelium, vimentin expression is negative. Conversely, it has been seen to show positive focal or diffuse expression in the endometrium’s stratum basalis and stratum functionalis during the menstrual cycle. This expression may follow cyclic variation with less expression during the secretory phase.

Hence, vimentin can be used as an indicator to differentiate between endometrial and endocervical adenocarcinoma; its interpretation should be included in a panel with other immunohistochemistry stains, particularly in situations when there is limited curetting and overlapping morphology^19^.

Regarding estrogen receptor, endometroid and mucinous endometrial adenocarcinoma shows strong and diffuse nuclear immunostaining while endocervical adenocarcinoma shows negative or weak focal immunostaining. It was also noted that the grade of endocervical adenocarcinoma is inversely related to the positivity of estrogen receptor immunostaining^15^. Progesterone receptor is less frequently seen in mucinous endometrial adenocarcinoma compared to endometroid endometrial adenocarcinoma.

In most cases of usual type endocervical adenocarcinoma, carcinoembryonic antigen (CEA) shows diffuse positivity with cytoplasmic and luminal border. While CEA shows weak and luminal positivity in endometrial adenocarcinoma especially if polyclonal CEA is used^13^. Other less frequently markers could be used as CD34 and CD10. Negative CD10 and positive CD34 staining of stromal cells favoring the diagnosis of endocervical adenocarcinoma^7^.

Previous literature reports focusing primarily on cervical SCC with endometrial and fallopian tubes spread while unusual spread of cervical adenocarcinoma has rarely been reported^8^. Table 1 provides a summary of reported cases of cervical adenocarcinoma and cervical adenocarcinoma in situ with unusual spread to the endometrium in the past 10 years.

**Table 1:** A summary of reported cases of cervical adenocarcinoma and cervical adenocarcinoma in situ with unusual spread to the endometrium in the past 10 years.

Case No.	Age (years)	Clinical presentation/impression	Prehysterectomy diagnostic specimen	Gross findings	Tumor type	Cervical tumor component	Uterine corpus tumor component	Additional pathological findings	Immunohistochemistry notes
1^3^	82	Endometrial cancer	Grade 1 endometroid endometrial adenocarcinoma	Grossly unremarkable	Usual type	Adenocarcinoma in situ (AIS) measuring 16 mm, spread horizontally up to the low uterine segment, no stromal invasion	Endometrium: multiple foci of intramucosal adenocarcinoma. Myometrium: no invasion	–	P16: strong positive ER: negative PR: negative
2^8^	50	Abnormal Pap smear, atypical squamous cell and atypical glandular cells.	Well differentiated adenocarcinoma of the cervix with mucinous and intestinal features	-Red glandular lesion in the cervix measuring 3.5×3.5 cm. -Shaggy endometrium measuring up to 0.4 cm in thickness	Endocervical type with focal mucinous and intestinal differentiation	Invasive adenocarcinoma, 7 mm invasion with surface extension to the upper genital tract.	Endometrium: intramucosal adenocarcinoma Myometrium: no invasion	Focal noninvasive involvement of the right fallopian tube	P16: diffuse positive ER: negative Vimentin: negative Ck20: negative P53: negative HPV type: 18
3^20^	36	Abnormal Pap smear. Endocervical curettage revealed intestinal type endocervical adenocarcinoma with cervical intraepithelial neoplasia (CIN) 2	Intestinal type endocervical adenocarcinoma in situ	Grossly unremarkable	Intestinal type endocervical adenocarcinoma in situ	Extensive cervical adenocarcinoma in situ.	Endometrium: intramucosal adenocarcinoma. Myometrium: no invasion	Diffuse invasion in both tubes	P16: strong positive ER: negative CEA: positive HPV types: 16,45
4^21^	66	Hydrometra	Leiomyosarcoma and atypical endometrial epithelial lesion	1.5 cm brownish mass in the cervix invaded the stroma with an irregular border. The lumen of the corpus was expanded, and the mucosa was diffusely irregular	Mainly gastric-type cells and partly of intestinal type	Adenocarcinoma in situ (AIS)	Endometrium: intramucosal adenocarcinoma with stromal invasion in continuous and skip patterns	synchronous cervical leiomyosarcoma	P16: negative Vimentin: positive P53: focal positive

## Conclusion

This case illustrates the possibility for misdiagnosing cervical adenocarcinoma as endometrial adenocarcinoma, particularly in cases when the lesion is in the lower uterine region and while analyzing curettage tissues. In these situations, the original tumor’s origin should be determined by combining the histologically observed morphological features with the immunohistochemical results. The rarity of this unusual spread of cervical adenocarcinoma accompanied with its challenges in diagnosis highlight the importance of reporting it to add to the existing scientific knowledge of this entity.

## Ethical approval

This study is exempt from ethical approval in our institution ‘Al-Quds University Ethics Committee’ as the patient consent was taken and authors have no conflict of interest.

## Consent

Written informed consent was obtained from the patient for publication and any accompanying images. A copy of the written consent is available for review by the Editor-in-Chief of this journal on request.

## Source of funding

The authors declare that writing and publishing this manuscript was not funded by any organization.

## Author contribution

D.J.: data collection; D.J., Z.M.M.Z., S.B., and S.J.: study concept or design and review and editing the manuscript; D.J., Z.M.M.Z., S.B., and S.J.: writing the manuscript; S.B.: histopathological interpretation.

## Conflicts of interest disclosure

The authors declare that there is no conflict of interest regarding the publication of this article.

## Research registration unique identifying number (UIN)

1. Name of the registry: not applicable.
2. Unique identifying number or registration ID: not applicable.
3. Hyperlink to your specific registration (must be publicly accessible and will be checked): not applicable.

## Guarantor

Dr Saadah Jaber.

## Data availability statement

Dataset is available upon reasonable request.

## Provenance and peer review

Not commissioned, externally peer-reviewed.
